# Endemicity of *Acinetobacter calcoaceticus-baumannii* Complex in an Intensive Care Unit in Malaysia

**DOI:** 10.1155/2015/789265

**Published:** 2015-12-27

**Authors:** Amreeta Dhanoa, Ganeswrie Rajasekaram, Soo Sum Lean, Yuet Meng Cheong, Kwai Lin Thong

**Affiliations:** ^1^Jeffrey Cheah School of Medicine and Health Sciences, Monash University Malaysia, 47500 Bandar Sunway, Malaysia; ^2^Department of Pathology, Hospital Sultanah Aminah Johor Bahru, 80100 Johor Bahru, Malaysia; ^3^Institute of Biological Sciences, Faculty of Science, University of Malaya, 50603 Kuala Lumpur, Malaysia

## Abstract

*Introduction*.* Acinetobacter calcoaceticus-baumannii* complex (ACB complex) is a leading opportunistic pathogen in intensive care units (ICUs). Effective control of spread requires understanding of its epidemiological relatedness. This study aims to determine the genetic relatedness and antibiotic susceptibilities of ACB complex in an ICU in Malaysia.* Methodology*. Pulsed field gel electrophoresis (PFGE), E-test, and disk diffusion were used for isolates characterization.* Results*. During the study period (December 2011 to June 2012), 1023 patients were admitted to the ICU and 44 ACB complex (blood, *n* = 21, and blind bronchial aspirates, *n* = 23) were recovered from 38 ICU patients. Six isolates were from non-ICU patients. Of the 44 ICU isolates, 88.6% exhibited multidrug-resistant (MDR) patterns. There was high degree of resistance, with minimum inhibitory concentration_90_ (MIC_90_) of >32 *μ*g/mL for carbapenems and ≥256 *μ*g/mL for amikacin, ampicillin/sulbactam, and cefoperazone/sulbactam. Isolates from the main PFGE cluster were highly resistant. There was evidence of dissemination in non-ICU wards.* Conclusion*. High number of clonally related MDR ACB complex was found. While the ICU is a likely reservoir facilitating transmission, importation from other wards may be important contributor. Early identification of strain relatedness and implementation of infection control measures are necessary to prevent further spread.

## 1. Introduction

The genus* Acinetobacter* comprises Gram-negative, strictly aerobic, nonmotile, non-lactose-fermenting, oxidase-negative, catalase-positive coccobacilli [1]. Members of the* Acinetobacter calcoaceticus-baumannii* complex (ACB complex) [2, 3] are the predominant* Acinetobacter* in clinical settings. Healthcare-associated infections caused by* Acinetobacter baumannii* are increasingly seen among immunocompromised populations and frequently cause outbreaks [1–3].* Acinetobacter* spp. are among the most common isolates from intensive care units (ICUs) in most Malaysian hospitals [4]. The challenge of managing infections caused by* Acinetobacter baumannii* has been complicated by the emergence and spread of multidrug-resistance (MDR), with both endemic and epidemic occurrences [1, 2]. Hospital acquired pneumonia associated with* Acinetobacter* spp. in Asian countries showed very high rate of resistance to imipenem at 67.3%, with especially high rates in Malaysia (86.7%), Thailand (81.4%), India (85.7%), and China (58.9%) [5].

In addition to the selection pressure exerted by widespread antibiotics usage, increased spread of MDR* Acinetobacter baumannii* may result from transmissions of resistant strains via contaminated surfaces, objects, and colonized healthcare workers [1–3, 6–8]. It is believed that infection reflects only the tip of an iceberg, with colonization reflecting the submerged portion [3, 9]. From an epidemiological perspective, it is useful to determine the relatedness (clonality) of these organisms, especially in endemic situations and epidemic outbreaks. If these strains are predominantly clonally related, improved compliance with infection control measures will be necessary [6, 8, 10].

Genotyping of nosocomial* A. baumannii* is useful to assess cases of cross-infections or to identify the sources and modes of spread of the organisms. These genotypic methods include pulsed field gel electrophoresis (PFGE) [11], PCR-based fingerprinting, amplified fragment length polymorphism, multilocus sequence typing (MLST) [12], and whole genome sequencing [13]. Among these, PFGE is the most widely used method for strain typing to determine the genetic relatedness of nosocomial strains [11].

This study attempts to understand the epidemiology of ACB complex isolated from patients in the ICU by determining the genetic relatedness and antimicrobial susceptibility patterns of these isolates.

## 2. Methodology

### 2.1. Study Population

This is a retrospective study examining the molecular epidemiology and antibiotic susceptibilities of clinical ACB complex conducted at a 989-bed tertiary care hospital in Johor, Malaysia, and it involved patients admitted to the ICU. Patients from whom ACB complex was isolated from blood and blind bronchial aspirates (BBAs) from December 2011 to June 2012 following admission to one of the two adult ICUs (West and South) were included in the study. The West ICU is located on the first floor of the hospital and has 22 beds: 14 beds with 1 : 1 nurse-to-patient ratio and 8 beds with 1 : 2 ratio. The South ICU is located on the second floor of the hospital and has 7 beds, all with 1 : 1 nurse-to-patient ratio. The infection control practices follow the consensus statement of infection control measures in ICU by the Intensive Care Section of the Malaysian Society of Anaesthesiologists (2009).

A total of 44 clinical isolates of ACB complex were isolated from ICU during the study period. In addition, 6 non-ICU isolates (blood, *n* = 4, and BBA, *n* = 2) were available and were included in the PFGE typing to determine whether they were clonally related to the ICU strains.

### 2.2. Clinical Data

Clinical data was extracted from the case report forms of the ICU patients with ACB complex cultured from blood and BBA. The following data were obtained from these patients: demographic characteristics, possible predisposing factors, prior and current antibiotic use, duration of ICU stay (Figure 3), clinical outcome, and whether the isolated organism was regarded as causing an infection or colonization. Clinical data was not retrieved for the 6 non-ICU patients.

ACB complex strains were regarded as colonizers based on the clinical judgement (clinical signs, laboratory parameters, and chest radiology findings) of the attending doctor. Invasive device as a risk factor was defined as having a device or invasive procedure for at least 48 hours within the 2 weeks preceding the date of the positive cultures. Prior antibiotic exposure was defined as the use of a systemic antibiotic agent for at least 72 hours within the 2 weeks preceding the date of the positive culture. Multidrug-resistant (MDR) ACB complex was defined as an isolate that was not susceptible (intermediately resistant or resistant) to three or more antimicrobials classes [14].

### 2.3. Bacterial Identification and Antimicrobial Susceptibility Testing

All blood cultures were processed by the clinical microbiology laboratory using the BACTEC 9240 system (Becton Dickinson and Company, Franklin Lakes, NJ, USA). ACB complex was identified by standard microbiological techniques (nonmotile, strictly aerobic, Gram-negative coccobacilli, catalase-positive and oxidase-negative, and inability to ferment glucose) and API 20 NE (bioMérieux, Marcy-l'Étoile, France).

Antibiotic susceptibility was performed using a panel of 11 different antimicrobial agents and was determined by means of Kirby-Bauer disk diffusion method using the guidelines provided by Clinical Laboratory Standard Institute (CLSI, 2012) [15]. The ACB complex isolates were tested against ampicillin/sulbactam (10/10 *μ*g), imipenem (10 *μ*g), meropenem (10 *μ*g), gentamicin (10 *μ*g), amikacin (30 *μ*g), ceftazidime (30 *μ*g), cefepime (30 *μ*g), piperacillin/tazobactam (100/10 *μ*g), cefoperazone/sulbactam (30/75 *μ*g), ciprofloxacin (5 *μ*g), and colistin (10 *μ*g) (Oxoid, Ltd., Basingstoke, Hampshire, England).

The E-test was used to determine the minimal inhibitory concentrations (MIC) of ampicillin/sulbactam, doripenem, meropenem, amikacin, cefoperazone/sulbactam, and colistin (AB BIODISK, Solna, Sweden) against ACB complex based on CLSI guidelines [15]. Since there are no CLSI interpretation criteria relevant to ACB complex for cefoperazone/sulbactam, the susceptibility breakpoints for these antibiotics were based on the MIC interpretive standards of CLSI for Enterobacteriaceae [15].* Escherichia coli* ATCC 35218 and* Pseudomonas aeruginosa* ATCC 27853 were used as quality control strains.

### 2.4. Molecular Typing Using PFGE

Molecular subtyping of the 50 ACB complex isolates was carried out using pulsed field gel electrophoresis (PFGE), as previously described [16]. Briefly, chromosomal DNA of these ACB complex isolates was prepared in agarose gel blocks, followed by digestion using* Apa*I restriction enzyme (Promega, Madison, WI, USA). Then, restricted DNA fragments were separated using CHEF Mapper (Bio-Rad, Hercules, CA, USA). Gels were run at 14°C in 0.5x TBE buffer for 24 hours, with pulse time of 2–40 seconds. The* Xba*I-digested* Salmonella enterica* ser. Braenderup H9812 was used as the molecular marker in these runs. Cluster analysis of the PFGE profiles was based on the Dice coefficient of similarity analyzed by BioNumerics 6.0 software (Applied Maths, Kortrijk, Belgium) using the Unweighted Pair Group Method with Arithmetic Averages (UPGMA) algorithm at 1.5% band position tolerance. Only DNA bands within the molecular marker (20.5 kb–1135 kb) were scored.

### 2.5. Ethical Approval

The study was approved by the Malaysian Ministry of Health Medical Research Ethics Committee (NMRR-13-288-14809). Informed consent was not obtained from the patients, as this was a retrospective study and the data was collected after patients had been discharged.

## 3. Results

### 3.1. Clinical Characteristics

During the 7-month period of the study, 1023 patients were admitted to the two adult ICUs and a total of 44 ACB complex positive cultures (blood, *n* = 21, and BBA, *n* = 23) from 38 ICU patients were identified. The clinical data from 35 patients was available for analysis (Table 1). The median patient age was 46 years (range: 14 to 73 years) and 65.7% of patients were males. All patients were ventilated and had central venous catheters. The median number of invasive devices was 3 (ranging from 2 to 5). Almost all patients (94.3%) had received antibiotics within 2 weeks of the positive ACB complex culture, the most common being broad-spectrum penicillins (65.7%), carbapenems (45.7%), and broad-spectrum cephalosporins (37.1%). Twenty-two (63%) patients had received ≤2 antibiotics and 11 (31.4%) had received ≥3 antibiotics. However, the number of antibiotics prior to culture positivity was not significantly higher among patients with MDR ACB complex (*P* = 0.565). The most common diagnoses upon admission to the ICU were motor vehicle accident with polytrauma (*n* = 8 patients). The mean (±SD) Acute Physiology and Chronic Health Evaluation (APACHE) II score was 23.2 (±7.0), with no significant difference between the MDR and non-MDR groups (*P* = 0.781). The most frequent comorbidities in this study were diabetes mellitus (25.7%) and hypertension (22.9%). The median time that had elapsed between admission and isolation of ACB complex was 5 days (range: 0 to 38 days); in 4 patients, ACB complex was recovered within the first 48 hours after admission. The median length of ICU stay was 13 days (range: 3 to 46 days). While all the bacteraemic cultures were regarded as clinically significant, all the BBA isolates were deemed to be colonizers. Overall mortality was 57% (20 out of 35 patients); 17 patients died while being in ICU and 3 died after being transferred out of ICU. While mortality was associated with underlying disease in 16 patients, in 4 patients it was believed to be directly related to ACB complex sepsis. These 4 patients were treated with polymyxin B and were severely compromised because of the underlying condition; first patient had severe burns, 2 patients had multiple injuries sustained in a motor vehicle accident, and the fourth patient had cancer of ovary. Despite the administration of appropriate antibiotic therapy, these patients succumbed to their illness.

Overall, polymyxin B was used to treat ACB complex infections in 11 patients and cefoperazone/sulbactam was used in 2 patients. In 18 patients, antibiotics were not administered as these BBA cultures were regarded as clinically nonsignificant, whereas in 4 patients antibiotics were not commenced as laboratory results were available only after the patients had died.

### 3.2. Antimicrobial Susceptibility

The antibiotic susceptibility pattern (disk diffusion) of the 44 ACB complex isolates as presented in Table 2 showed that majority (>80%) of the isolates were nonsusceptible (resistant and intermediately susceptible) to carbapenems, ciprofloxacin, piperacillin/tazobactam, ceftazidime, cefepime, cefoperazone/sulbactam, and ampicillin/sulbactam. Slightly better results were demonstrated with the aminoglycosides (nonsusceptibility rates of 79.5% for gentamicin and 72.7% for amikacin). Of the 44 ICU isolates, 88.6% exhibited MDR patterns. All strains were susceptible to colistin. Results of the MIC of selected antibiotics are shown in Table 3. MIC_50_ and MIC_90_ for both doripenem and meropenem were 32 *μ*g/mL or higher, suggesting high degree of resistance to carbapenems in this collection. Most isolates were highly resistant to amikacin with MIC_50_ and MIC_90_ of >256 *μ*g/mL. Twenty-two (50%) isolates were resistant to cefoperazone/sulbactam with 14 (31.8%) demonstrating intermediate susceptibility. Ampicillin/sulbactam, another commonly used antibiotic, demonstrated higher resistance compared to cefoperazone/sulbactam: 72.7% (resistant) and 13.6% (intermediate susceptibility). Extremely high MIC value of 256 *μ*g/mL was required to inhibit 90% of ACB complex with ampicillin/sulbactam, cefoperazone/sulbactam, and amikacin. The BBA isolates had higher MIC_90_ for ampicillin/sulbactam and cefoperazone/sulbactam compared to blood isolates, although not significantly different. The MIC distribution of colistin was as follows: 0.125 *μ*g/mL (*n* = 1), 0.190 *μ*g/mL (*n* = 3), 0.250 *μ*g/mL (*n* = 23), 0.380 *μ*g/mL (*n* = 7), 0.5 *μ*g/mL (*n* = 9), and 1 *μ*g/mL (*n* = 1). The cut-off MIC values for colistin are as follows: sensitive, ≤ 2 *μ*g/mL, intermediate, 4 *μ*g/mL, and resistant, ≥ 8 *μ*g/mL.

### 3.3. Genetic Diversity

**Figure 1:**
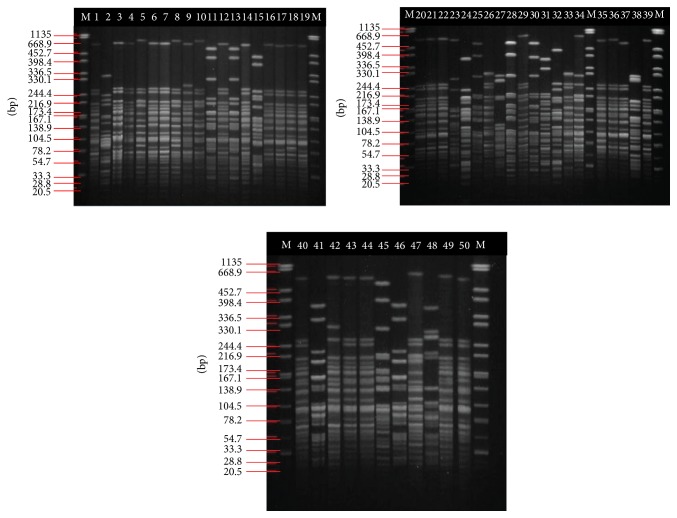
Pulsotypes of 50 clinical ACB complex isolates from a tertiary hospital in Malaysia. Lane M represents* Salmonella* ser. Braenderup H9812 as the marker. Lanes 1–50 represent clinical isolates JBAC01-JBAC50.

**Figure 2:**
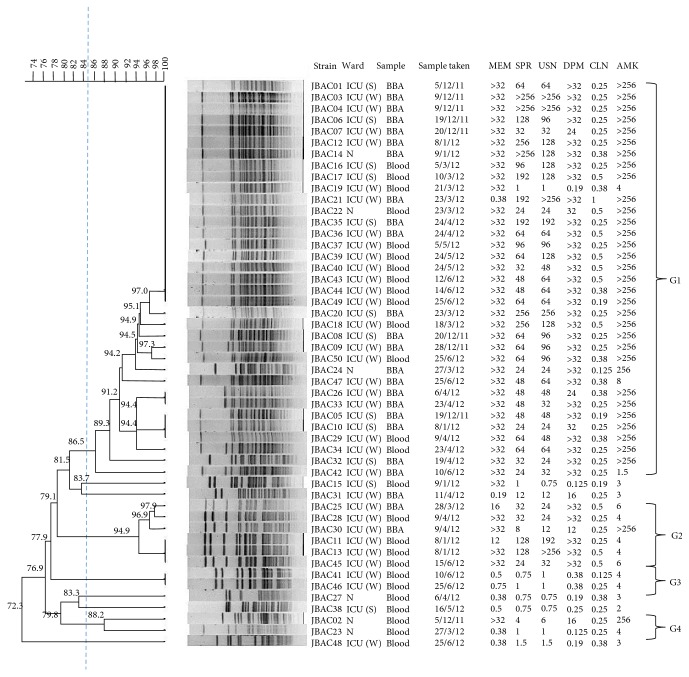
Dendrogram of the PFGE profiles of* Apa*I-digested 50 ACB complex isolates using Unweighted Pair Group Method with Arithmetic Averages (UPGMA). The dotted vertical line indicates the cut-off point of 85% similarity. The different clusters at 85% similarity are arbitrarily designated G1–G4, whereby G1 is the largest group representing the most prevalent pulsotypes and its variants among the isolates.

**Figure 3:**
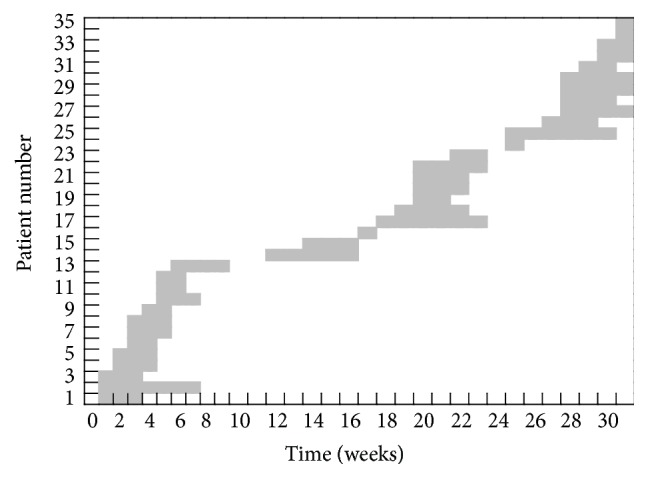
Timeline for ICU stay for patients infected/colonized with ACB complex, December, 2011, to June, 2012.

PFGE separation of* Apa*I-digested chromosomal DNA of 50 isolates (ICU = 44 and non-ICU = 6) generated 25 distinct, reproducible patterns (pulsotypes) (Figure 1). A dendrogram based on all the pulsotypes was generated (Figure 2). On the basis of 85% similarity, four clusters, G1 to G4, were observed (Figure 2). The major cluster, G1, consisted of 35 isolates with closely related pulsotypes (less than 4-band difference). A predominant endemic pulsotype was shared among 18 ICU and three non-ICU isolates. Twenty isolates (blood and BBA samples) isolated throughout the study period from ICU and non-ICU wards were indistinguishable, indicating their endemicity. The G1 cluster consisted of isolates from both ICUs (West and South) and included 4 patients whose cultures (blood = 2 and BBA = 2) were taken within 48 hours of ICU admission. This isolate was also recovered from 3 patients who were not admitted to either of the 2 ICUs. Except for 5 isolates, cluster G1 contained isolates that were highly resistant to carbapenems (MIC ≥ 32 *μ*g/mL) and amikacin (MIC ≥ 256 *μ*g/mL) (Figure 2). Similarly, majority of the isolates were resistant to ampicillin/sulbactam and cefoperazone/sulbactam, although the MICs were more variable.

In contrast to cluster G1 isolates, which were recovered from West and South ICUs and the non-ICU wards, the 6 isolates within cluster G2 were recovered exclusively from West ICU. Unlike cluster G1, these isolates were mostly susceptible to amikacin (except strain JBAC30). Within this cluster, two isolates (JBAC11 and JBAC13) isolated from same patient from different body sites had identical unique pulsotype.

Cluster G3 consisted of two isolates with a unique pulsotype recovered from West ICU. These blood isolates which were recovered 15 days apart from the same patient were identical but distinctly different from the other* A. baumannii* (with more than 5-6-band difference), indicating persistent infection in the same individual.

Cluster G4 comprised non-ICU isolates recovered from blood and they were more variable, indicating the discriminatory power of PFGE. Except for JBAC02, the other isolates (JBAC23) in cluster G4 were sensitive to the tested antibiotics.

By and large, vast majority (70%) of the MDR isolates belonged to G1 cluster and 14.3% of them belonged to G2 cluster. No MDR isolates were found in G3 and G4 clusters.

## 4. Discussion

In this study, PFGE was proved to be a discriminative genotyping tool. We found evidence of a dominant genotype (cluster G1) of MDR ACB complex in vast majority (74%) of the isolates. These isolates were recovered from patients cared for in both ICUs (West and South) throughout the 7-month period of the study, raising serious concerns about the persistence and resilience of this epidemic strain. The high genetic relatedness of these isolates suggests cross-transmission within the ICU setting. Four isolates belonging to cluster G1 were identified within 48 hours of ICU admission from non-ICU wards, supporting the probable rate of transmission from the non-ICU wards. However, the 48-hour criterion for ICU acquisition should be interpreted with some caution, as epidemic strains have been acquired within 48 hours of ICU admission [17].

There was also evidence of dissemination of this dominant genotype in the non-ICU wards, as 3 out of 6 randomly collected non-ICU blood isolates showed similar genotype. Based on this, we can assume that this particular genotype of ACB complex might not be confined to the ICU wards alone but may actually be found in other wards in the hospital. Nevertheless, although transfer from non-ICU wards was a likely contributory factor, the ICU itself is a probable reservoir facilitating transmission as the resistant clone seemed endemic and persistent in the ICU. In a study conducted in another tertiary teaching hospital in Malaysia, Kong et al. [16] demonstrated that this organism had established endemicity in that hospital as isolates from the environment and hands of healthcare workers and patients were indistinguishable. The striking similarity among the isolates suggests cross-transmission which may occur by direct contact by infected or colonized person or indirectly through environmental contamination, medical devices, or healthcare workers [1–3, 6–8]. In the present study, the role of environmental source could not be adequately investigated partly because we did not have a complete collection of the isolates. However,* A. baumannii* is able to survive for long periods of time on dry surfaces and this tolerability to desiccation contributes to its persistence in hospital environments and transmission through fomites [1, 10]. Wilks et al. [8] reported a recent outbreak of MDR* Acinetobacter* infection with environmental contamination found on curtains, laryngoscope blades, patient lifting equipment, door handles, mops, and keyboards.

Outbreaks caused by MDR ACB complex have been reported in several ICUs worldwide [6–8]. Analysis of resistance determinants and genetic relatedness demonstrated that widespread dissemination of a highly resistant dominant G1 cluster had contributed to the high prevalence of MDR ACB complex in the ICU (88.6%). The second most prevalent cluster (cluster G2) comprised 12% of the isolates and prevailed primarily in the West ICU. The isolates within this cluster were also highly resistant, except that unlike the isolates in cluster G1 they remain sensitive to amikacin. Two other minor clusters (G3 and G4) comprising mainly sensitive isolates also coexisted during the study period. This concurred with another study by Lean et al. [11], whereby multiple subtypes (pulsotypes < 4-band difference) of* A. baumannii* were shown to exist in another tertiary hospital in Malaysia.

Overall, PFGE revealed that the isolates were genetically related, which is typical of outbreak episodes. Nevertheless, PFGE was discriminative enough to differentiate unrelated isolates as seen in 3 out of the 6 non-ICU isolates which had unique profiles (cluster G4) and 2 other isolates taken from the same patient 15 days apart within G3 cluster. However, because of the endemicity of the predominant ACB complex cluster, more discriminative methods, other than PFGE, such as whole genome sequencing (WGS) may be needed to pinpoint the source of transmission routes of very closely related ICU strains. WGS provides the ultimate strain differentiation and has been successfully applied to investigate nosocomial outbreaks caused by* A. baumannii* [13], MRSA [18, 19], and* Klebsiella pneumoniae* [20].

Concurring with other studies [9, 21], the highly immunocompromised nature of our patients predisposed them to serious ACB complex infection as evidenced by 21 episodes of bacteraemia. Although the 23 BBA cultures were deemed to be colonizers clinically, identification of colonizers becomes equally important as colonization may serve as an exogenous source of infection and colonization for other patients and may be a risk factor for subsequent endogenous infections [2, 22]. Since majority of the isolates were clonally related, emphasis should be placed on preventing acquisition and transmission of this infection [6, 10]. Once endemic in a healthcare setting,* A. baumannii* or ACB complex can be extremely challenging to eliminate. Numerous outbreaks of MDR* A. baumannii* have been documented in Asian countries. An* A. baumannii* outbreak in an ICU in Taiwan was interrupted through the institution of cohort nursing of patients, improved hand hygiene, and effective environmental and equipment decontamination [22]. Active surveillance and environmental cleaning resulted in sustained reduction of MDR* A. baumannii* colonization and infection rates and reduced the cost of antibiotics usage and hospitalization among ICU patients in Thailand [9]. Another such outbreak in Singapore was ceased only after the ICU was closed for complete cleaning, although initial control measures which included screening of all patients and immediate isolation and cohorting of patients with contact precautions helped in containing the outbreak [23].

## 5. Conclusions

PFGE analysis revealed that the ACB complex isolated from the ICU patients belongs mainly to one major cluster. While the ICU itself is a likely reservoir facilitating transmission, importation of strains from other wards of the hospital may be an important contributory factor. However, regardless of the original source of the outbreak strain and whether it was subsequently transmitted from human or environmental source, early identification and close scrutiny together with prompt implementation of multidisciplinary infection control interventions are required to prevent further spread in the ICU.

## Figures and Tables

**Table 1:** Clinical characteristics of 35 patients with ACB Complex.

Characteristics	*N* (%)/median (range)
Age (years)^*∗*^	46 (14–73)
Length of ICU stay (days)^*∗*^	13 (3–46)
Gender (male)	23 (65.7)
Bacteraemia	17 (48.6)
APACHE score^*∗*^	22 (14–44)
Diabetes mellitus	9 (25.7)
Hypertension	8 (22.9)
Malignancy	3 (8.6)
Renal disease	5 (14.3)
Cardiac disorder	3 (8.6)
Respiratory disorder	3 (8.6)
Neutropenia	2 (5.7)
Polytrauma	8 (22.9)
Respiratory disorder	3 (8.6)
Prior antibiotic therapy	33 (94.3)
ES cephalosporins	13 (37.1)
Broad-spectrum penicillin	23 (65.7)
Quinolones	1 (2.9)
Carbapenem	16 (45.7)
Aminoglycosides	3 (8.6)
Metronidazole	4 (11.4)
Macrolide	4 (11.4)
Cotrimoxazole	2 (5.7)
Number of prescribed antibiotics^*∗*^	2 (0–6)
Number of invasive procedures/devices^*∗*^	3 (2–5)
Central venous catheter	35 (100)
Endotracheal tube	35 (100)
Urinary catheter	28 (80)
Dialysis catheter	15 (42.9)
Drainage	3 (8.6)
Prior surgical procedure	3 (8.6)
ICU outcome (died)	17 (48.6)

All characteristics are expressed as number (%), except *∗* which is expressed as median (range).

**Table 2:** Antimicrobial susceptibility (disc diffusion) of 44 ACB complex isolates recovered from 38 ICU patients.

Antibiotics	Nonsusceptible
	(intermediate and resistant) (%)
GEN	35 (79.5)
AKN	32 (72.7)
IMI	39 (88.6)
MER	39 (88.6)
CIP	37 (84.1)
PZ	39 (88.6)
CAZ	38 (86.4)
FEP	39 (88.6)
AMS	37 (84.1)
CPS	37 (84.1)
COL	0

GEN, gentamicin; AKN, amikacin; IMI, imipenem; MER, meropenem; CIP, ciprofloxacin; PZ, piperacillin/tazobactam; CAZ, ceftazidime; FEP, cefepime; AMS, ampicillin/sulbactam; CPS, cefoperazone/sulbactam; COL, colistin.

*Antimicrobial categories*: aminoglycosides (gentamicin and amikacin); antipseudomonal carbapenems (imipenem and meropenem); antipseudomonal fluoroquinolones (ciprofloxacin); antipseudomonal penicillin + *β*-lactamase inhibitors (piperacillin/tazobactam); extended-spectrum cephalosporins (ceftazidime and cefepime); penicillins+ *β*-lactamase inhibitors (ampicillin/sulbactam), cephalosporin+ *β*-lactamase inhibitors (cefoperazone/sulbactam); polymyxin (colistin).

*MDR*: nonsusceptible to ≥1 agent in ≥3 antimicrobial categories.

**Table 3:** MIC (*μ*g/mL) for 44 ACB complex isolates recovered from 38 ICU patients.

Antibiotic	*S* (%)	*I* (%)	*R* (%)	MIC_50_			MIC_90_		
				All	B	BBA	All	B	BBA
AMS	6 (13.6)	6 (13.6)	32 (72.7)	64	64	64	256	128	>256
DOR	6 (13.6)	0 (0)	38 (86.4)	>32	>32	>32	>32	>32	>32
MER	6 (13.6)	0 (0)	38 (86.4)	>32	>32	>32	>32	>32	>32
AKN	14 (31.8)	0 (0)	30 (68.2)	>256	>256	>256	>256	>256	>256
CPS	8 (18.2)	14 (31.8)	22 (50)	48	48	64	256	128	256
COL	44 (100)	0 (0)	0 (0)	0.25	0.38	0.25	0.5	0.5	0.5

B, blood; BBA, bronchoscopic aspirate; AMS, ampicillin/sulbactam; DOR, doripenem; MER, meropenem; AKN, amikacin; CPS, cefoperazone/sulbactam; COL, colistin.
